# Improving the Diagnosis of Autoimmune Gastritis: From Parietal Cell Antibodies to H+/K+ ATPase Antibodies

**DOI:** 10.3390/diagnostics14161721

**Published:** 2024-08-08

**Authors:** Michela Tonegato, Maria Piera Panozzo, Antonio Antico, Nicola Bizzaro

**Affiliations:** 1 Department of Laboratory Medicine, AULSS2 Marca Trevigiana, 31100 Treviso, Italy; michelatonegato@gmail.com (M.T.); antonio.antico@aulss2.veneto.it (A.A.); 2 Department of Laboratory Medicine, AULSS7 Pedemontana, 36061 Santorso, Italy; mariapiera.panozzo@aulss7.veneto.it; 3 Laboratory of Clinical Pathology, Azienda Sanitaria Universitaria Integrata, 33100 Udine, Italy

**Keywords:** parietal cell autoantibodies, autoimmune gastritis, chronic atrophic gastritis, H+/K+-ATPase, immunoassays

## Abstract

Parietal cell autoantibodies (PCAs), which recognize the enzyme H+/K+-ATPase as a target, are considered to be a diagnostic marker of autoimmune gastritis and pernicious anemia; these conditions are characterized by the presence of corpus atrophic gastritis. Circulating PCAs can be detected using several analytical methods that are commonly available in the clinical laboratory. Traditionally, indirect immunofluorescence (IIF) on rodent or primate stomach tissue is used as a screening test for the detection of PCAs. However, IIF suffers from a high inter-observer variability and lacks standardization. In addition, like immunoblotting, results are expressed only in a qualitative or semi-quantitative manner. Based on the few available studies that are reviewed herein, quantitative enzyme-linked immunosorbent assays (ELISAs) and fluorescence enzyme immunoassays (FEIAs) using purified H+/K+-ATPase perform better than IIF in the detection of PCAs, displaying higher sensitivity and utility in monitoring the disease. In light of their higher diagnostic accuracy, these solid-phase methods should be preferred to IIF in the screening of autoimmune atrophic gastritis. The use of methods to detect antibodies versus a specific subunit of H+/K+-ATPase (α or β) is currently confined to the world of research. Further investigation is required to define the clinical utility of H+/K+-ATPase subunit detection.

## 1. Introduction

Chronic atrophic autoimmune gastritis (CAAG) is an organ-specific autoimmune condition that is mainly characterized by the progressive destruction of the gastric body and fundus glands [1]. The disease is estimated to affect between 0.1% and 1–2% of the general population; this prevalence rises to 2.5–3% in individuals over 60 years of age, with a female/male ratio of 2–3:1 [2]. However, estimating the prevalence of CAAG is highly dependent on the diagnostic criteria used (serological, clinical, or endoscopic/histological), which must be appropriately integrated [2,3].

The key pathogenic mechanism is represented by the activation of an inflammatory response that can lead to the destruction of the native gastric glands (parietal and zymogenic cells) with the subsequent and progressive development of intestinal metaplasia, atrophy, and hyperplasia of enterochromaffin-like cells [4,5]. This condition may evolve into a gastric carcinoid tumor or an adenocarcinoma in approximately 10% of cases [6,7,8,9]. The cellular degenerative process results from the interaction of T lymphocytes (especially CD4+ and, to a lesser extent, CD8+), T regulatory cells, B lymphocytes, macrophages and natural killer cells with proteins that are secreted by gastric parietal cells and with proteins that are located on their surface [4]. The strict interactions between these cells and the secretion of cytokines from families of interleukins (IL), interferons, and growth factors allow the inflammatory response to be maintained and immune reactions to be induced.

In particular, T helper1 (Th1) cells may promote the death of gastric epithelial cells through the activation of the Fas-Fas ligand and perforin/granzyme B cytotoxic pathways mediated by Th1 cytokines (interferon-gamma, IL-2 and TNF-α). On the other hand, T helper 17 (Th17) cells play a crucial role in tissue damage and in the loss of gastric mucosal parietal cells, contributing to the progressive destruction of gastric glands. They secrete IL-17 family cytokines, which promote the self-immune response towards H+/K+ ATPase mediated by CD4+ T cell; this finding can explain the pathogenicity of Th17 cells in CAAG, denying the long-held belief that this condition is mediated exclusively by Th1 [10]. A recent study demonstrated that IL-17A and IL-17F were produced in vivo in the stomachs of CAAG patients following activation with H+/K+-ATPase; moreover, serum IL-17A, IL-17F, IL-21, and IL-17E levels were significantly elevated in CAAG patients, but not in those without CAAG [11]. These results suggest that the measurement of IL-17 family cytokines might be useful not only for the management of CAAG patients, but also for predicting the development of gastric cancer in patients with gastric atrophy [12].

Chronic T-cell-dependent B cell activation determines the in situ production of autoantibodies that are directed towards those cells of the corpus–fundus mucosa in which the inflammatory processes had taken place [1]. Therefore, this event seems to be responsible for the local production of antibodies to intrinsic factors and to gastric parietal cells (PCAs). The recognition of PCAs by gastric T cells, in turn, stimulates the secretion of other Th1 and Th17 cytokines in a self-maintaining loop. 

Parietal cells are epithelial cells that are located in the glands of the corpus and fundus, but not in the antrum; for this reason, CAAG atrophy is limited to the mucosa of the gastric body [13,14]. In this part of the stomach, the destruction of parietal cells leads to a reduced gastric acidity and a loss of intrinsic factor, which, in turn, determine iron and B12 vitamin malabsorption, resulting either in microcytic iron-deficient anemia or in macrocytic vitamin B_12_-deficient anemia (pernicious anemia). The former may be present in up to 25–50% of patients with CAAG, whereas the latter may be found in up to 15–25% of patients [3,15].

The key–lock mechanism was first described in an experimental study conducted in 1987; this study allowed the identification of the autoantigen [16]. In this work, in 75–95% of cases, antibodies from patients with CAAG were able to recognize a particular cytoplasm component of the gastric parietal cells that was obtained after high-speed centrifugation and separation on a saccharose–Ficoll gel. This component was identified in the transmembrane protein H+/K+-adenosine triphosphatase (H+/K+-ATPase) [16]. This enzyme, whose function is to promote the secretion of H+ ions into the gastric lumen in exchange for K+ ions that are internalized in the gastric parietal cell, is the main antigenic target of PCAs [13,17].

H+/K+-ATPase consists of four subunits, two α chains (100 kDa) and two β chains (60–90 kDa), constituting a transmembrane protein arranged at the level of the intracellular secretory channels of gastric parietal cells [18,19]. α subunits contain the catalytic site and mediate ion transport, while β subunits play a fundamental role in stabilizing the α subunits and are critical for enzyme function [20,21]. The human H+/K+-ATPase β-subunit comprises a 33 kD core protein with seven possible N-linked glycosylation sites. Both α and β chains are required for the ATPase activity of ion transport across the parietal cell membrane. Early scientific evidence has suggested that in autoimmune responses, PCAs react predominantly toward highly glycosylated β subunits, which are able to trigger an autoimmune reaction by themselves. Later, it was highlighted that this reactivity extends to other epitopes by also involving the α subunit in binding to the antibody [22,23]. It has been demonstrated that the removal of these parts from the β-subunit of pig H+/K+-ATPase eliminated autoantibody binding; on the other hand, disulfide bonds in the β-subunit were essential for gastric H+/K+-ATPase activity and also for autoantibody recognition [18]. 

## 2. Clinical Meaning of PCA

Over the past two decades, increased interest in the pathogenesis and evolution of CAAG has led to the search for serological markers that are able to detect possible changes in the gastric mucosa at an early stage [5,24,25]. The most recent reports emphasize how the combination of pepsinogen I, pepsinogen II, and PCA serum levels can provide useful information in suspecting a gastric abnormality, whereby a reduced pepsinogen I/pepsinogen II ratio is indicative of functional atrophic impairment, whereas a positivity for PCAs suggests the autoimmune nature of the gastric alteration [26,27,28]. Maastricht VI guidelines underline the utility of these serological markers in providing clinically useful information on the likelihood of gastric mucosal atrophy, including its etiology [9]. A position paper produced by the Gastroenterological and Endoscopic Italian Scientific Societies reinforces the following assumption: a combination of gastric functional biomarker (pepsinogens, G-17, and anti-*Helicobacter pylori* antibodies) serum assays seems to be the most reliable non-invasive screening tool to select those patients to be referred for an endoscopy, among those at an increased risk for chronic atrophic gastritis. In this consensus, the presence of PCAs was recognized as a diagnostic hallmark for CAAG, even if strict diagnostic criteria for this condition are still missing [14].

PCAs are positive in about 80% of patients with CAAG [24], as well as in 85–90% of patients with pernicious anemia [4]. However, a subset of patients may be seronegative. Conti reported a significant inverse correlation between age and autoimmune gastritis and PCA titer [29]; additionally, seronegative CAAG is more frequent in older patients, in particular in those over 70 years of age [4]. This finding could be explained by the natural history of CAAG, whereby the concentration of PCAs tends to increase, peak, and gradually decline from early stage to late-stage gastritis, until they totally disappear; this occurs alongside the progressive destruction of gastric mucosa and the consequent loss of the antigenic target [30,31]. Therefore, the absence of autoantibodies does not definitively exclude the possibility of autoimmune atrophy. 

It should be noted that PCAs are not restricted to CAAG; these antibodies may be present in other autoimmune diseases [32,33]. Hashimoto’s thyroiditis is a frequent finding in patients with CAAG (with a prevalence of 20–30%), and, in turn, patients with autoimmune thyroid diseases are frequently affected by a gastric autoimmune atrophic condition. The prevalence of PCA positivity in patients with autoimmune thyroid diseases seems similar in both sexes and is not age-dependent [4]. It has been demonstrated that the presence of PCAs in patients with an autoimmune thyroid disease carries a greater risk for developing CAAG compared to PCA-positive subjects without autoimmune thyroid disease [32]. Given the close link between autoimmune thyroid disease and PCA positivity, the scientific community suggests that high-risk patients should be serologically screened for PCAs in order to investigate a hidden or initial CAAG condition. On the other hand, CAAG patients should be studied for a likely undiagnosed autoimmune thyropathy.

Serum PCAs are also frequently associated with type 1 diabetes mellitus (13–20% in adults and 5% in children), with a 3- to 5-fold increase in CAAG onset compared to controls. In addition, these autoantibodies more frequently present in patients with type 1 diabetes than in their first-degree relatives, suggesting that the diabetic condition alone plays an important role. In multiple autoimmune syndrome, CAAG occurs in 15% of type 3 (in association with diabetes mellitus and autoimmune thyroid disease) and in 10–15% of type 1 (hypoparathyroidism, Addison’s disease, diabetes mellitus, and mucocutaneous candidiasis) diseases. PCAs are also found among individuals with vitiligo (15%), celiac disease, alopecia, myasthenia gravis, connective tissue diseases, and autoimmune hepatitis [5]. These associations may be explained by the presence of similar genetic HLA haplotypes and common phenotypic pathogenetic mechanisms. 

Finally, PCAs can be found, although with an unclear significance, in 2.5–9% of healthy adults who will never develop CAAG or pernicious anemia [4]. This PCA prevalence, which derives from screening procedures on healthy individuals, varies depending on the assay used, as demonstrated by Bagnasco et al. [34]. 

The presence of PCAs alone is not sufficient to diagnos CAAG, as a histological finding of gastric body atrophy is required. However, it is important to consider that PCAs may precede the appearance of atrophic lesions of the stomach by several years [29,35].

## 3. Analytical Methods for Serum PCA Determination

The present work provides an overview, through an examination of the scientific literature, of the main analytical techniques currently used in autoimmunology laboratories for PCA determination to define their analytical performance and to assess their usefulness in the diagnosis and follow-up of patients affected by CAAG.

PCAs can be detected using many analytical methods (indirect immunofluorescence [IIF], enzyme-linked immunosorbent assays [ELISAs], fluorescence enzyme immunoassays [FEIAs], and immunoblotting [IB]). We did not find any studies that used the radioimmunological method, which is now unused in diagnostic laboratories, or the chemiluminescence immunoassay (CLIA). However, CLIA is an analytical method with great development potential [36] and it is foreseeable that within a few years many companies producing diagnostic tests will introduce automated CLIA tests for PCAs.

To carry out comparative analyses among methods, special attention was paid to scientific papers describing the analytical principles and instrumental platforms used, as well as the unit of measurement, cutoff, sensitivity, and specificity.

**Figure 1:**
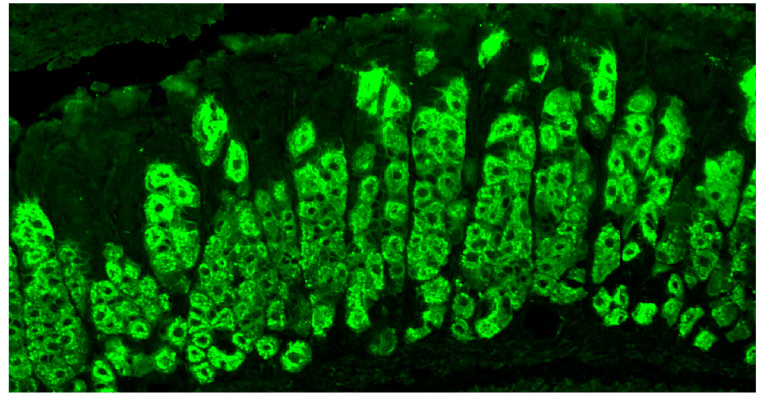
Positive fluorescence pattern for antibodies against gastric parietal cells (PCAs) obtained after incubation of the cell substrate (mouse stomach section) with serum from a patient with chronic atrophic autoimmune gastritis. The typical picture is indicated by diffuse fluorescence in gastric parietal cell cytoplasm, while the nuclei are negative. Also negative are the zymogenic cells (indirect immunofluorescence 1:40, 400×).

Traditionally, PCA screening tests are based on the IIF technique on rodent or monkey stomach tissue sections [35,37,38]. After serum incubation with the substrate, the addition of a fluorescein-labeled secondary antibody allows for the highlighting of the antigen–antibody reaction, which will be more evident with a higher autoantibody concentration [4]. The typical picture is indicated by a diffuse fluorescence in the cytoplasm of gastric parietal cells (Figure 1) [37,38,39]. Serial serum dilutions allow PCA titer estimation.

Unfortunately, this methodological approach has some limitations. For example, fluorescence reading is time-consuming, requires an experienced operator, and is affected by a high inter-observer variability [32,37]. Moreover, interpretations can be challenged when anti-mitochondrial antibodies are present, which can obscure the cytoplasmic staining of gastric parietal cells [39]. In addition to the limitations mentioned above, the lack of standardization is evident; in studies where tests were performed using IIF, different dilution titers were established as cutoffs for positivity (1:10, 1:20, or 1:40) [39,40,41]. Furthermore, only in two studies was the endpoint dilution described to provide the antibody concentration [34,38]. 

ELISAs, FEIAs, and IB are the solid-based assays (SPAs) that are most widely used for the detection of H+/K+ ATPase antibodies. These methods may differ depending on the animal antigenic source (porcine, murine, ovine, or primate) and the class of immunoglobulins. ELISAs provide a quantitative measurement of serum PCAs; this approach is useful for highlighting changes in PCA levels during CAAG progression [37]. Antigen–autoantibody binding can be revealed in a microplate by the addition of a peroxidase-conjugated anti-human IgG antibody, resulting in a chromogenic reaction that is detected as a change in absorbance [16]. Due to its higher sensitivity [40] and the automation of the analytical run, this method should be preferred to IIF [24,35,37] However, its main limitation lies in the need to dispose of a minimum number of samples to set up a batch-based session. In recent years, analytical methods that allow random instrumental access at any time during routine activity have been gradually replacing the traditional ELISA. These include FEIAs, whose analytical principle is based on the use of H+/K+-ATPase, which is purified from porcine gastric mucosa [35,40] and a fluorescent molecule as a tracer. Compared with ELISAs, FEIAs are easier to perform and it is possible to continuously load samples onto the analyzer with a reduced analysis and reporting time [42].

Regarding IB, the solid phase is represented by a nitrocellulose membrane onto which the purified H+/K+ ATPase antigen is fixed. An antibody provided with a chromogenic substrate binds the antigen–antibody complex, resulting in a colorimetric reaction whose intensity, detected using optical densitometry, is proportional to the serum autoantibody concentration [40]. The main limits of this analytical principle are represented by qualitative and/or semiquantitative results, as well as by the complexity of the work session set-up when an automatized system is not available.

Clinical pathologists often struggle with the choice of analytical method, having to consider the diagnostic accuracy of each method, resources, and personnel availability, as well as his/her own experience. In this scenario, the clinician is faced with interpreting results for the same immunological markers obtained using different methods, which is affected by analytical variability and differences in inter-laboratory approaches. 

It should be emphasized that none of the available methods represents the gold standard for PCA assessment [4,40]. Aside from IIF, published data on the performance of solid-phase assays for PCAs are relatively poor and are concentrated in the last few years, in which solid-phase assays were introduced.

In one study, comparing the analytical performance of IIF and ELISAs for PCA detection in a group of subjects undergoing routine diagnostic workup, the ELISA showed a higher sensitivity than IIF, without a significant loss of specificity; this was likely due to the different characteristics of the antigen used (rodent tissue for IIF, at a serum dilution of 1:40, and pig purified for ELISA) [36]. Therefore, the authors recommended the ELISA method in place of IIF because of its better diagnostic performance, which allowed PCA detection at an earlier stage of CAAG.

Later, other works confirmed this assumption. Bagnasco et al. [34] demonstrated not only a good analytical sensitivity of ELISA in IIF-negative samples (considering two substrates—rodent stomach at 1:40 serum dilution and monkey stomach at 1:10 serum dilution), but also its excellent specificity. The good performance of ELISAs for PCA detection was demonstrated either for the early diagnosis of CAAG or for monitoring its evolution towards atrophy [33].

A recent Dutch study [40] compared the performance of several commercially available PCA detection methods for the first time. In total, seven immunoassays were analyzed, based on four methods (two IIF, three ELISAs, and one each for FEIA and IB) from different manufacturers. The comparative analysis was performed considering different cohorts of patients, as follows: a control group of healthy elderly subjects, patients with an active infection, patients randomly selected from a population with a request for PCA testing irrespective of the results, and biobank sera that tested positive for PCAs. As noted above, in the absence of a reference gold standard for PCA detection, the authors calculated Cohen’s kappa for each of the methods tested against a consensus result, which was defined as the number of matching results for five out of the seven assays tested (either positive or negative, with equivocal results counted as positive). The sensitivity and specificity of the different assays were calculated based on the unanimous result (positive or negative) obtained from six out of the seven tests considered. The authors used two different substrates (rat versus monkey stomach), showing good agreement between these two IIF tests (Cohen’s kappa 0.8 to 1.0), with an excellent sensitivity (99% and 95%) and specificity (99% for both) (Table 1); this is consistent with results from another study [34]. The three ELISA tests showed a better performance than IIF, with a higher sensitivity (always 100%). This is important evidence when considering that PCAs may already be present in asymptomatic patients with initial CAAG [35]. Only one ELISA provided a slightly lower specificity (96% compared with 99–100% from the other two), due to some false positives in the group of patients with active infection. The FEIA showed an excellent analytical performance in terms of sensitivity and specificity (100%), both higher than IIF. As regards IB, a lower sensitivity (95%) was found due to the difficulties in interpreting the results around the cutoff value. The authors concluded that IB, per se, seems less suitable for PCA detection than the other methods [40].

Overall, a comparison of the different methods made in this study led the authors to state that most of the assays showed a good diagnostic performance and that the differences among them may be related to the different antigenic sources. However, the study cited shows two limits: 1. the lack of clinical data underestimates the accuracy of the diagnostic sensitivity and specificity, and 2. the lack of an analytical gold standard for PCAs does not allow for the resolution of borderline results. Therefore, low-positive or borderline results should be interpreted with caution [40].

## 4. H+/K+-ATPase Subunits in CAAG Diagnosis and Monitoring

Commercial enzyme immunoassays for PCAs do not distinguish which proton pump subunit is recognized by the antibodies. The α subunit (ATP4A) is believed to be the main antigen, while the β subunit (ATP4B) allows for the stabilization of the ATP4A subunit [20,43]. Lahner’s group developed an assay based on the Luciferase Immunoprecipitation System (LIPS), targeting the ATP4A and ATP4B subunits of ATP4 to define the role of each subunit in the disease’s progression [20]. LIPS is based on cloning the sequences coding for ATP4A and ATP4B into vectors; these chimeric antigens are expressed by the polymerase chain reaction amplification of luciferase-conjugated probes that are detected using a luminometer.

Increased ATP4A and ATP4B antibody levels were found in patients with PCAs and in subjects with a clinical suspicion of gastric body atrophy (presence of dyspepsia, anemia, and histological findings of a concomitant *Helicobacter pylori* infection). A correlation between ATP4B positivity and the presence of severe atrophy of the gastric body and intestinal metaplasia was also observed [43]. Gastric proton pump subunits detected using LIPS, either separately or combined, showed a higher sensitivity compared to the total PCAs assayed using an ELISA [20]. The sensitivity of LIPS was 100% for both ATP4A and ATP4B, and the specificity was 89% and 90% for ATP4A and ATP4B, respectively. On the other hand, in this study, the sensitivity of ELISA was 69% and the specificity was 91%. Furthermore, the ATP4B subunit, which is strongly involved in autoantibody recognition, was the serological biomarker with the best individual performance [20,43]. The differences between LIPS and ELISA could be explained by some technical aspects. For example, LIPS can detect both conformational and linear epitopes through the use of human recombinant antigens that are labeled with a highly active luciferase reporter, while ELISA often shows the suboptimal detection of conformational epitopes [43]. These studies confirm that the H+/K+ ATPase antibody test, regardless of whether the method recognizes the entire molecule or its subunits, is a promising tool for screening patients with suspected CAAG for endoscopy referral and histological examination.

## 5. Discussion

In recent years there has been an ongoing debate to evaluate whether the IIF method can still reasonably be considered as the reference method for the detection of many autoantibodies, or whether it should be replaced by more specific, reproducible, and automated immunometric methods. This question concerns the research of anti-nuclear antibodies on HEp-2 cells, of anti-neutrophil cytoplasmic antibodies, and of all the autoantibodies that are detected on triple rodent tissue, such as anti-mitochondria, anti-smooth muscle, anti-liver–kidney–microsomal (LKM), and PCAs [44]. 

A national survey conducted by the Study Group on Autoimmunology of the Italian Society of Clinical Pathology and Laboratory Medicine in 2019 showed that 86.5% of the 123 participating laboratories use IIF for PCA determination, 5.2% use only ELISA or FEIA, and 8.3% use both IIF and SPA [45]. These data are not restricted to Italian laboratories, as similar data can be observed by analyzing the use of immunologic methods for laboratories across the world who participate in the UK-NEQAS external quality assessment programs for PCAs [46]. This finding, showing that IIF is still the preferred method for searching for PCAs, is surprising considering the technical and diagnostic limitations of this assay. It should be pointed out, however, that it is affected by the decades-long established use of IIF, and by the fact that, currently, many laboratories only have the IIF method available for detecting PCAs.

The standardization of the IIF technique remains a challenge as different commercial methods use different antigenic substrates and disagree in proposing uniform screening dilutions. Furthermore, IIF expresses results only in semi-quantitative terms (antibody titer), so it does not appear a reliable method for disease follow-up. Though guidelines on this antibody test are lacking, scientific evidence reiterates the need for a routine automated immunoassay that is more reproducible and has a higher diagnostic accuracy. These requirements, which are not present in IIF and IB, are largely met by ELISAs and FEIAs. Their characteristic of being quantitative tests allows their use not only to help diagnose CAAG, but also to monitor the evolution of the disease. Indeed, antibody levels may decrease and even disappear, along with the progressive destruction of gastric mucosa and the consequent loss of the antigenic target.

However, although ELISA represents a robust method, there is no consensus on the definition of the cutoff, nor on the sensitivity and specificity values, which vary depending on the assay manufacturer. Currently, compared with the ELISA microplate, FEIAs seem to better fulfill the demand for automation and random instrumental access.

When CAAG is suspected, the diagnostic algorithm traditionally involves IIF as a screening test that is followed, when available, by a confirmatory solid-phase test. However, given the increasing number of PCA test requests addressed to the clinical laboratories and the increasing use of automated solid-phase assays, with their price containment, they can be proposed as the preferred screening method for PCA detection. In the case of uncertain or borderline results, it might be appropriate to confirm the result with a second method (i.e., IIF or IB). 

Finally, it should be noted that the currently available ELISA and FEIA immunoassays do not discriminate regarding the reactivity of antibodies directed toward a specific gastric proton pump subunit. Identifying whether autoantibodies target one subunit (α or β) or both could be an important breakthrough not only for the early diagnosis of CAAG, but also for assessing the clinical meaning of seropositivity in asymptomatic people, for the differential diagnosis of chronic atrophic gastritis and risk stratification. In order to not remain confined to basic science, this field of investigation requires further studies aimed at obtaining a reliable test for routine diagnostics and for the follow-up of patients with CAAG.

## Figures and Tables

**Table 1:** Diagnostic accuracy of assays for PCAs (modified from ref. [40]).

Method	Antigen Source	Cutoff	Sensitivity (%)	Specificity (%)
IIF (Inova Diagnostics, San Diego, CA, USA)	Rat stomach	1:20	98.7	98.8
IIF (Euroimmun, Lübeck, Germany)	Monkey stomach	1:10	95.1	98.8
ELISA (Orgentec, Mainz, Germany)	Porcine	10 U/mL	100	96.3
ELISA (Euroimmun, Lübeck, Germany)	Porcine	≥1 (ratio)	100	98.8
ELISA (Inova Diagnostics, San Diego, CA, USA)	Porcine	25 U/mL	100	100
IB (D-tek, Mons, Belgium)	Porcine	10 AU	95.1	100
FEIA (Thermo Fisher, Freiburg, Germany)	Porcine	10 U/mL	100	100

IIF, indirect immunofluorescence; ELISA, enzyme-linked immunosorbent assay; IB, immunoblot; FEIA, fluorescence enzyme immunoassay; AU, arbitrary units.
